# New Advertisers and Subscribers

**Published:** 1919-05

**Authors:** 


### New Advertisers.

The Journal presents a number of new advertisers to its readers this month and asks consideration of their claims to your patronage. The New York Intravenous Laboratory has a two page advertisement that cannot escape the attention of any one. The Abbott Laboratories, of Chicago, have a most attractive page announcement. The Eureka Springs Commercial Club uses a half page to tell of the advantages of that mountain watering place, The Nucleoids Company, of Chicago, uses a quarter page to advertise its Leuchorrhea Special No. 9. Bristol-Myers Co., of Brooklyn, N. Y., take an eighth page for presenting their well-known Sal Hepatica.

The sanitariums are also well represented in the new advertisements, Battle Creek, Mich., using a page. Greenwood's Sanitarium at Houston for nervous and mental diseases, alcohol and drug addicts, have a half page announcement. Dr. Cloud's Oak Sanitarium at Austin for nervous and mental cases and liquor and drug addicts uses a third of a page. Kramer's Mudlavia at Kramer, Indiana, has a quarter page announcement of its mud bath treatment. The Sector Sanitarium at Kerrville has a quarter page presenting the claims of that mountain sanitarium resort. Two new cards appear in this issue—that of Dr. E. H. Trowbridge of Kansas City, and that of Dr. J. Spencer Davis of Dallas.

In calling attention of the profession to these new advertises, the Journal would not have you overlook one of the old, for every advertising page of this publication is full of interest and helpfulness.

---

### New Subscriptions This Month.

The Texas Medical Journal acknowledges the receipt of the following subscriptions received since the last issue, all of which are paid a year in advance: Dr. J. H. Ogle, Garland, Texas; Dr. P. W. Weeks, Onalaska, Texas; Dr. J. W. Falvey, Longview, Texas; Dr. A. E. Johns, Palacios, Texas; Dr. H. S. Eargle, Burnet, Texas; Dr. Stephen E. Smith, Crobyton, Texas; Dr. J. G. McLaurin, Dallas, Texas; Dr. O. R. Marshall, Moody, Texas; Dr. R. E. Taylor, Alpine, Texas; Dr. J. C. Hill, Hallville, Texas; Dr. T. E. McGarity, Coma, Texas; Dr. W. J. Patton, Blossom, Texas; Dr. D. W. Holmes, Belleveue, Texas; Dr. A. F. Mathews, Brownwell, Texas; Dr. Ivison Grimes, Camden, Texas; Dr. G. G. Kemper, Emory, Texas; Dr. J. S. Lipscomb, Crow, Texas; Dr. L. A. Nixon, El Paso, Texas; Dr. James J. Cappleman, Honey Grove, Texas; Dr. W. D. Black, Barstow, Texas; Dr. J. S. Taylor, Cookville, Texas; Dr. R. P. Knolle, Ellinger, Texas; Dr. L. E. Cockerell, Eustace, Texas; Dr. W. W. McGregor, Laredo, Texas; Dr. H. H. McConnaghy, Houston, Texas; Dr. W. F. Whittenberg, Honey Grove, Texas; Dr. T. C. Dye, Addison, Texas; Dr. E. E. McGowan, Paducah, Texas; Dr. S. G. Norris, Chicota, Texas.

